# 

**Published:** 1906-09-01

**Authors:** 


### NOTICE TO CORRESPONDENTS.

All MSS., Letters, Books for Review, and other matters intended for the Editor should be addressed The Editor, "The Hospital," The Hospital Buildings, 28 & 29 Southampton Street, Strand, W.C.

The Editor cannot undertake to return rejected MS., even when accompanied by stamped directed envelope.

### Notice to Advertisers and Subscribers.

All Advertisements, Orders for Copies of The Hospital, and Business Communications should be addressed to **THE MANAGER (Not to the Editor),** The Hospital, 28 & 29 Southampton Street, Strand, London, W.C.

The Cost of Subscription, post free, is as follows:—Per week, 3½d.; per quarter, 3s. 3d.; per half-year, 6s. 6d.; per annum, 13s. Foreign and Colonial:—Per annum, 19s. 6d., payable, in all cases, in advance.

**ART OF FEEDING THE INVALID.**

By a MEDICAL PRACTITIONER and a LADY PROFESSOR OF COOKERY.

New and Popular Edition, with Special Chapter on Feeding in Consumption. 1/6 post free.

**DIET IN SICKNESS AND IN HEALTH.**

By Mrs. ERNEST HART. With an Introduction by Sir HENRY THOMPSON, F.R.C.S., M.B., Lond. 3/6 post free.

**ELEMENTARY TREATISE ON THE LIGHT TREATMENT FOR NURSES.**

By JAMES H. SEQUEIRA, M.D. Lond. Illustrated. 2/6 net, 2/9 post free.

**HINTS TO NURSES ON TROPICAL FEVERS.**

By S. F. POLLARD, Sister Army Nursing Reserve, late Sister Guy's Hospital.

The Work is based upon the Personal Experience of the Author Abroad. 1/6 net, 1/8 post free.

**INSTRUCTIONS TO MIDWIVES.**

Compiled by the Midwives Supervising Committee of the Corporation of Manchester. 6d. net, 7d. post free.

**LECTURES ON HOME NURSING FOR THE POOR.**

By a DISTRICT NURSE. Illustrated. 1/6 net, 1/8 post free.

**LECTURES UPON THE NURSING OF INFECTIOUS DISEASES.**

By F. J. WOOLLACOTT, M.A., M.D. 2/6 net, 2/9 post free.

**MEDICAL ELECTRICITY AND THE LIGHT TREATMENT.**

By KATE NEALE, Sister in Charge Light Department, Guy's Hospital. Illustrated. 2/6 net, 2/9 post free.

**NURSING IN DISEASES OF THE THROAT, NOSE AND EAR.**

By P. MACLEOD YEARSLEY, F.R.C.S. Eng., M.R.C.S., L.R.C.P. Lond. Illustrated. 2/6 post free.

**OPHTHALMIC NURSING.**

By SYDNEY STEPHENSON, M.B., F.R.C.S.E.

New and Revised Edition profusely Illustrated, and a Glossary of Terms. 3/6 net, 3/10 post free.

**OUTLINES OF INSANITY.**

**A Popular Treatise on the Salient Features of Insanity.**

By FRANCIS H. WALMSLEY, M.D. Popular Edition, stitched. 1/6 post free.

**SIMPLE INTRODUCTORY LESSONS ON MIDWIFERY.**

By M. LOANE. Illustrated. 1/- net, 1/1 post free.

**SIMPLE SANITATION:**

**The Practical Application of the Laws of Health to Small Dwellings.**

By M. LOANE. With Introductory Chapter on Sanitary Legislation by D. A. MEARNS PROSER. 1/- net, 1/2 post free.

**THE CARE OF CHILDREN.**

**Practical Hints for Mothers and Nurses at Home and Abroad.**

By ROBT. J. BLACKHAM, D.P.H. Lond., C.B. Revised and Enlarged Edition. 1/6 net, 1/8 post free.

**THE HUMAN BODY.**

**Its Personal Hygiene and Practical Physiology.**

By B. P. COLTON, Professor of Natural Science, Illinois University. Profusely Illustrated. 5/- post free.

**THE MODERN NURSING OF CONSUMPTION.**

By Dr. JANE H. WALKER. 1/- net, 1/2 post free.

☞ *Catalogue of Nursing Manuals sent post free on application.* ☜

**The Scientific Press, Ltd.,**

**Publishers of Nursing Text-Books, Charts, and Case-Books of all Descriptions.**

**28 & 29 SOUTHAMPTON STREET, STRAND, LONDON, W.C.**

**Publications.**

### A SELECTION OF
### CASSELL & COMPANY'S MEDICAL WORKS.

**A Manual of Operative Surgery.** By Sir FREDERICK TREVES, Bart., G.C.V.O., C.B., F.R.C.S., LL.D. Revised by the Author and JONATHAN HUTCHINSON, Jun., F.R.C.S. With about 450 Illustrations. Revised Edition. Complete in 2 vols., 42s. Supplied in sets only.

**A Manual of Chemistry:** Inorganic and Organic. Covering the Synopsis of the Conjoint Board and the Society of Apothecaries. By ARTHUR P. LUFF, M.D., B.Sc.Lond., F.R.C.P., F.I.C., and FREDERIC JAMES M. PAGE, B.Sc.Lond., F.I.C. With 43 Illustrations. New and Revised Edition. 7s. 6d.

**Hygiene and Public Health.** By B. ARTHUR WHITELEGGE, C.B., M.D., B.Sc. Lond., F.R.C.P., &c., and GEORGE NEWMAN, M.D., D.P.H., F.R.S.E., &c. Illustrated. New and Revised Edition. 7s. 6d.

**Materia Medica and Therapeutics.** An Introduction to the Rational Treatment of Disease. By J. MITCHELL BRUCE, M.A., LL.D. Aberd., M.D., F.R.C.P., &c. New, Revised, and Enlarged Edition. 7s. 6d.

**Clinical Methods:** A Guide to the Practical Study of Medicine. By ROBT. HUTCHISON, M.D., F.R.C.P., and HARRY RAINY, M.A., M.D., F.R.C.P.Edin., F.R.S.E. With 9 Coloured Plates and upwards of 150 Illustrations. New and Revised Edition. 10s. 6d.

**First Lines in Midwifery.** A Guide to Attendance on Natural Labour for Medical Students and Midwives. By G. ERNEST HERMAN, M.B. Lond., F.R.C.P. With numerous Illustrations. 5s.

**A Manual of Medical Treatment.** By I. BURNEY YEO, M.D., F.R.C.P., Consulting Physician to King's College Hospital, &c. 2 vols. 21s. net.

**The Student's Handbook of Surgical Operations.** By Sir FREDERICK TREVES, Bart., G.C.V.O., F.R.C.S., &c., and JONATHAN HUTCHINSON, Jun., F.R.C.S. With about 120 Illustrations. New Edition. 7s. 6d.

**Surgical Applied Anatomy.** By Sir FREDERICK TREVES, Bart., G.C.V.O., C.B., F.R.C.S., &c., &c. Revised with the assistance of ARTHUR KEITH, M.D., F.R.C.S. With 80 Engravings. 9s.

**Elements of Surgical Diagnosis:** A Manual for the Wards. By A. PEARCE GOULD, M.S., M.B., F.R.C.S. New and Enlarged Edition. 9s.

**A Handbook for Midwives and Maternity Nurses.** By COMYNS BERKELEY, B.A., B.C. Cantab., M.R.C.P., M.R.C.S., Assistant Obstetric Physician to the Middlesex Hospital. With 59 Illustrations. 5s.

**** A Complete List of Medical Works will be sent post free on application.*

CASSELL & CO., LIMITED, London, Paris, New York, and Melbourne.

### CLEFT PALATE AND HARE-LIP:
**The Earlier Operation on the Palate.**

By **EDMUND OWEN**, M.B., F.R.C.S., Consulting Surgeon of St. Mary's Hospital, and to the Hospital for Sick Children, Great Ormond Street, London.

Pp. 111, with 32 Illustrations. Just Published. Price **2s. 6d.** net.

"A most valuable contribution to the literature of this subject."—*British Medical Journal.*

London: BAILLIÈRE, TINDALL, & COX, 8 Henrietta Street, W.C.

JUST PUBLISHED. Price **2s. 6d.** net.

### CARCINOMA OF THE RECTUM.

By F. SWINFORD EDWARDS, F.R.C.S.,

Surgeon to the West London Hospital; Senior Surgeon to St. Peter's Hospital for Stone, and to St. Mark's Hospital for Fistula, &c.

London: Baillière, Tindall, & Cox, 8 Henrietta Street, Covent Garden.

THE MOST EFFECTUAL BANDAGE FOR THE CURE OF ULCERS AND OTHER DISEASES OF THE LEG.

Now Ready.—All Booksellers.
Crown 8vo. 320 Pages. Cloth, Price 3s. 6d. Twenty-one Illustrations.

### THE RISE AND PROGRESS OF HYDROPATHY

IN ENGLAND AND SCOTLAND. By Richard Metcalfe, The Hydro, Richmond, Surrey, Author of "Essays and Notes on Hydro-therapeutics," "Life of Vincent Priessnitz," &c.
London: Simpkin, Marshall & Co.

A LIST of the NEW PUBLICATIONS of the SCIENTIFIC PRESS, LIMITED, will be sent post free to any member of the Medical Profession on receipt of post card.

28 & 29 SOUTHAMPTON STREET, STRAND LONDON, W.C.

